# Comparison of Dilution, Filtration, and Microwave Digestion Sample Pretreatments in Elemental Profiling of Wine by ICP-MS

**DOI:** 10.3390/molecules22101609

**Published:** 2017-09-25

**Authors:** Joshua Godshaw, Helene Hopfer, Jenny Nelson, Susan E. Ebeler

**Affiliations:** 1 Department of Viticulture & Enology, University of California, One Shields Ave, Davis, CA 95616, USA; jgodshaw@ucdavis.edu (J.G.); jenny_nelson@agilent.com (J.N.); 2 Food Safety & Measurement Facility, University of California, One Shields Ave, Davis, CA 95616, USA; 3 Department of Food Science, The Pennsylvania State University, 202 Food Science Building, University Park, PA 16802, USA; hopfer@psu.edu; 4 Agilent Technologies, 5301 Stevens Creek Blvd, Santa Clara, CA 95051, USA

**Keywords:** ICP-MS, sample preparation, wine analysis, isotope dilution, elemental profiling

## Abstract

Wine elemental composition varies by cultivar, geographic origin, viticultural and enological practices, and is often used for authenticity validation. Elemental analysis of wine by Inductively Coupled Plasma Mass Spectrometry (ICP-MS) is challenging due to the potential for non-spectral interferences and plasma instability arising from organic matrix components. Sample preparation mitigates these interferences, however, conflicting recommendations of best practices in ICP-MS analysis of wine have been reported. This study compared direct dilution, microwave-assisted acid digestion, and two filtration sample pretreatments, acidification prior to filtration and filtration followed by acidification, in elemental profiling of one white and three red table wines by ICP-MS. Of 43 monitored isotopes, 37 varied by sample preparation method, with significantly higher results of 17 isotopes in the microwave-digested samples. Both filtration treatments resulted in lower results for 11 isotopes compared to the other methods. Finally, isotope dilution determination of copper based on natural abundances and the ^63^Cu:^65^Cu instrument response ratio agreed with external calibration and confirmed a significant sample preparation effect. Overall, microwave digestion did not compare favorably, and direct dilution was found to provide the best compromise between ease of use and result accuracy and precision, although all preparation strategies were able to differentiate the wines.

## 1. Introduction

The analysis of the elemental composition of wines is important for several reasons. Several elements, such as As, Cd, and Pb, can have adverse health effects in humans even at low concentrations (<100 µg/L), while others (i.e., Cu) are essential nutrients that only become toxic at levels greater than typically encountered in the human diet [1], or are of importance for wine stability or development [2]. In addition, wine elemental composition has been shown to vary with origin, grape variety, environmental conditions, and viticultural and winemaking practices [2,3,4,5,6]. This variation in the elemental profile of wines can be utilized to distinguish between wines of different geographic origin as a means of authenticity validation. However, sample preparation methods are inconsistent among studies and have been shown to impact the results, with no consensus of which method is more appropriate for the elemental profiling of wines for authenticity purposes [7,8].

Wine elemental analysis is usually performed using atomic absorption and emission spectroscopy techniques such as Flame Atomic Absorption/Emission Spectroscopy (FAAS and FAES) and Inductively Coupled Plasma Atomic Emission Spectroscopy (ICP-AES) or Mass Spectrometry (ICP-MS). ICP-MS reports of sub-parts per billion detection limits, multielement analysis capabilities, and a wide linear dynamic range have made it an often used technique for wine elemental analysis [9]. However, elemental determination in wine, via plasma sources, presents a challenge analytically due to the risk of non-spectral interferences by organic matrix components, primarily ethanol when present in concentrations from 7 to 20% [7,10]. As such, direct analysis of wine by ICP-MS can affect plasma conditions and sample transport thus causing variations in signal stability and intensity [9,10]. Modifications on the instrumental configurations, e.g., using a microwave desolvation chamber, employing an internal standard (IS) and/or collision cell technology in combination with matrix-matched calibration are used to mitigate the effects and allow for direct wine analysis by ICP-MS [8].

Several sample preparation methods have been employed for the elemental analysis of wines, including direct dilution by factors ranging from one to 10, UV-assisted digestion, and open or closed vessel acid digestion assisted by microwave radiation [4,6,7,8,9,10,11,12]. It is important to note that no single sample pretreatment is recommended for the elemental profiling of wines by governing bodies. For example, official methods of the International Organization for Vine & Wine (OIV) call for alcohol removal by evaporation in Fe and Zn analysis while suggesting simple dilution for Cu and Mg analysis [13]. A standard multielement method requires a twenty-fold dilution in 5% HNO_3_ along with external calibration for ICP-MS analysis but suggests microwave digestion using concentrated nitric acid be applied to wines containing more than 100 g/L of sugar, although no digestion procedure is provided [13]. While high dilution factors help to minimize matrix effects in ICP-MS analysis, this can limit the ability to measure trace and ultratrace elements.

Examination of the results obtained using different sample preparation methods yields conflicting recommendations of best practices in wine elemental analysis. For instance, Gonzalvez et al. [7] concluded microwave assisted acid digestion should be used for wine elemental analysis after comparing the elemental composition of twelve Spanish red wines measured by ICP-MS after direct dilution, open vessel acid digestion, and microwave assisted closed vessel acid digestion pretreatments. The authors found nearly congruent results between both acid digestion methods with the exception of Ti and Li, which exhibited higher concentrations in the microwave digested samples. They also found significant differences for Mg, Cr, Fe, and Ba concentrations when comparing acid digestion with direct dilution (and matrix-matched calibration standards) and attributed these differences to plasma interferences due to the presence of alcohol and organic compounds.

A less decisive study by Grindlay et al. [8] compared microwave assisted acid digestion to direct dilution of two white, one red, and one brandy wine samples by measuring fifteen elements by ICP-MS. The authors reported lower detection limits and reduced matrix effects on ICP-MS measurements when the wine samples were microwave digested prior to analysis. However, they also suggested that direct dilution employing an internal standard was adequate for elemental analysis of wines, noting it was more user friendly and time efficient. Similar conclusions have been made in the comparison of microwave-assisted digestion with direct dilution pretreatments in wine elemental analysis employing an internal standard to account for plasma variability [4,11,14].

The contradicting recommendations for sample preparation treatments in ICP-MS analysis substantiate an experiment to compare them once again. Previous reports comparing sample preparation strategies in wine ICP-MS analysis do not employ the technology of a collision/reaction cell that is commonly used in most modern ICP-MS instruments. Collision/reaction cell technology (CRC) has been demonstrated to reduce the effects of spectral interferences in elemental analysis of wine by ICP-MS and allowed for the application of lower dilution factors [15]. Notably, it was determined that CRC must be applied for ultratrace analysis of ^47^Ti and ^52^Cr in wine to account for spectral interferences, both of which are implicated in differences observed among sample preparation strategies of wine by ICP-MS analysis [7,8,15]. Recent work in wine elemental analysis by ICP-MS has taken advantage of CRC and thus it is applied in this study [6,12,15].

In addition to microwave assisted acid digestion (MW) and direct dilution (DD), the use of filtration in sample preparation procedures is assessed. Filtration in wine analysis has been previously applied to remove particulate matter after dilution with an acid solution [5] but has not been fully investigated as a sample preparation step for ICP-MS analysis. The use of filters could impact the determination of elements that are known to form complexes with polyphenols, polysaccharides, and proteins in wine conditions [16]. Copper, for example, has been demonstrated to form complexes with polyphenols and tannins in red wines and model wine solutions via fluorometric quenching experiments [17]. The stability of these organic metal species varies greatly with pH and thus two treatments were devised to investigate the use of filters in wine sample preparation for ICP-MS analysis: filtration of the wine followed by acidification (FA) and acidification followed by filtration (AF).

To assess the accuracy and precision of each sample preparation treatment, recovery of isotopically labeled ^65^Cu and ^206^Pb spikes in addition to multi-element spiking experiments are employed, as no certified reference material (CRM) for elemental analysis of wine is currently available. Approximate levels of copper and lead in wines of below 0.1–10 mg/L and <100 µg/L, respectively, as well as atomic mass were considered in the selection of these isotopes [18]. Wine copper and lead levels are often monitored to assess wine stability or to account for associated health risks and assess the impact of anthropogenic activities. In fact, wine copper concentration is limited to 0.5 mg/L in the United States and the OIV limits wine lead levels to a maximum of 0.15 mg/L [13,19]. Additionally, copper is used as a viticultural antifungal agent and can be added to wine after fermentation as copper sulfate, up to 6.0 mg/L, to remove undesirable sulfidic aroma compounds via precipitation [19]. However, effective removal of added copper as its precipitates is often not achieved by current filtration strategies which some have attributed to the small particle size of the precipitates [20,21]. This can cause increased copper concentration in the finished wine where stability issues could arise, further justifying the need for its routine analysis.

An advantage of using stable isotope standards for spiking experiments is the potential to measure isotope ratios. Wine Sr and B isotope ratios have been investigated previously as potential indicators of geographical origin [22,23]. However, proper isotope dilution in ICP-MS analysis of wine has only been applied for lead determination [14,24,25,26]. In isotope dilution, isotopically labeled standards serve as both the internal standard and calibrator, offering the advantage of exact matrix matching of each sample. Isotope dilution offers comparable accuracy and has been shown to improve measurement precision of Pb in wine compared to external calibration [14,24]. Properly developed isotope dilution methods rely on the use of established isotope ratios or the variation in isotope ratio of the natural sample once it is spiked. Ultimately, this allows for a single point calibration to be used in routine sample analysis, eliminating the need for separate calibration standards, thus, decreasing the length of the analytical run and improving sample throughput. To our knowledge, copper determination in wine by isotope dilution with ICP-MS has not been previously reported, and its feasibility was thus investigated.

Here, we aim to compare the accuracy, precision, and detection limits of 43 isotopes in four wines by ICP-MS utilizing four sample preparation treatments (FA, AF, DD, and MW) and employing spike recovery of ^65^Cu and ^206^Pb stable isotope spikes besides multi-element spiking experiments. Additionally, we undertake preliminary exploration of isotope dilution as a calibration strategy for copper determination in all wines by each sample pretreatments.

## 2. Results and Discussion

### 2.1. Detection Limits and Method Blanks

Instrumental detection limits calculated using matrix matched calibration blanks (4% Ethanol, 5% HNO_3_) differed slightly between the direct methods (DD, FA, AF) and microwave digestion (MW), with MW detection limits being higher for twenty-three of the forty-three isotopes measured, Table 1. Equivalent instrumental detection limits were found for ^93^Nb, ^125^Te, ^137^Ba, and ^147^Sm. Isotopes detected in the method blanks for each sample preparation treatment were evaluated as the mean (*n* = 3) and differed with respect to preparation method as shown in Table 1. It is clear from inspection of mean method blank concentrations that microwave digestion (MW) presents the greatest risk of sample contamination, as evidenced by noticeably higher results (>1 µg/L) for ^27^Al, ^47^Ti, ^52^Cr, ^55^Mn, ^59^Co, ^60^Ni, ^66^Zn, ^98^Mo, ^181^Ta, and ^182^W, while ^7^Li was found at levels near or exceeding 1 µg/L by all preparation methods. Two additional analytes, ^27^Al and ^52^Cr, exceeded 1 µg/L in DD method blanks, but were still at levels more than four times less than MW method blanks. The high levels observed in MW method blanks point to the possibility of reagent contamination as these samples were digested in concentrated ultrapure nitric acid as opposed to the use of a dilute acid solution in the direct methods. While more analytes were detected in DD blanks than in either filtration method on average, only ^27^Al, ^52^Cr, ^55^Mn, and ^60^Ni were detected at levels greater than 0.5 µg/L when not detected in AF and FA method blanks. Though we observe a background, many of the elements detected in method blanks are at levels at least an order of magnitude lower than typically measured in wine and thus small sample contamination may be inconsequential relative to the detection of these elements in wine [18]. For example, the greatest raw contamination was observed for ^55^Mn in MW method blanks, which exceeded 20 µg/L, but represents less than two percent of average ^55^Mn levels in the wines studied. Method blank concentrations of four elements, ^93^Nb, ^123^Sb, ^181^Ta, and ^182^W, were found to exceed average wine concentrations in MW blanks. Normally these values would be omitted from any further analysis, however for the purposes of distinguishing among sample preparation techniques they are included here.

### 2.2. Profiling of Wines

Multivariate Analysis of Variance (MANOVA) of the samples showed a significant sample preparation method effect was present. Subsequent univariate Analysis of Variance (ANOVA) of the samples revealed significant differences by sample preparation method (*p* ≤ 0.05) for 37 of the 43 isotopes included in this analysis: ^7^Li, ^27^Al, ^47^Ti, ^52^Cr, ^55^Mn, ^59^Co, ^60^Ni, ^63^Cu, ^65^Cu, ^66^Zn, ^75^As, ^78^Se, ^85^Rb, ^88^Sr, ^93^Nb, ^98^Mo, ^101^Ru, ^103^Rh, ^107^Ag, ^111^Cd, ^123^Sb, ^125^Te, ^133^Cs, ^137^Ba, ^141^Pr, ^146^Nd, ^147^Sm, ^15^3Eu, ^157^Gd, ^165^Ho, ^169^Tm, ^181^Ta, ^182^W, ^205^Tl, ^206^Pb, and ^208^Pb (Table A1).

Average wine concentrations of monitored isotopes ranged from 3 ng/L for some rare-earth elements to greater than 1 mg/L for ^55^Mn and ^85^Rb by all preparation methods, as seen in Table A1. Most notably, MW results were significantly higher than all other methods for seventeen isotopes (^27^Al, ^55^Mn, ^59^Co, ^65^Cu, ^66^Zn, ^93^Nb, ^98^Mo, ^103^Rh, ^107^Ag, ^123^Sb, ^125^Te, ^133^Cs, ^137^Ba, ^146^Nd, ^181^Ta, ^182^W, and ^206^Pb). This is not surprising as all of these isotopes were detected in the MW method blanks with the exception of ^146^Nd. Higher concentrations of some elements in MW samples have been reported previously, including significantly higher results for ^59^Co in comparison to a two-fold wine dilution [4]. Furthermore, MW samples exhibited the lowest level of precision when RSDs of detected isotopes of each wine and preparation method are averaged, with a mean RSD = 29.5% compared to less than 13–18% for AF, FA, and DD. These values are inflated due to the inclusion of some elements (e.g., ^101^Ru, ^103^Rh, ^107^Ag, and ^182^W) at levels near detection limits where measurements are more variable. A possible source of variation in MW samples could be varying levels of sample contamination as mentioned previously. The digestion procedure used here was similar to those used in other reports [8] and was the manufacturer recommended procedure for solid food matrix digestion. Therefore, it is unlikely that the microwave-assisted matrix degradation is variable among the liquid wine samples.

Only ^27^Al, ^55^Mn, ^60^Ni, ^66^Zn, ^75^As, ^78^Se, and ^101^Ru differed significantly on average between AF and FA treatments, suggesting minimal effect of acidification on overall profile determination. Surprisingly, average wine concentrations were found to be lower in AF samples than FA samples for each of these isotopes, contrary to our hypothesis that AF could yield higher results compared to FA due to the dissociation of metal complexes with a lowered pH. Both FA and AF treatments yielded significantly lower results than DD or MW for ^7^Li, ^59^Co, ^63^Cu, ^85^Rb, ^88^Sr, ^93^Nb, ^133^Cs, ^182^W, ^205^Tl, ^206^Pb, and ^208^Pb, possibly owing to analyte loss as a result of filtration. This effect has been demonstrated in attempts to recover copper additions to model wine, and wine to a lesser extent, using 0.45 µm filtration [20].

**Figure 1:**
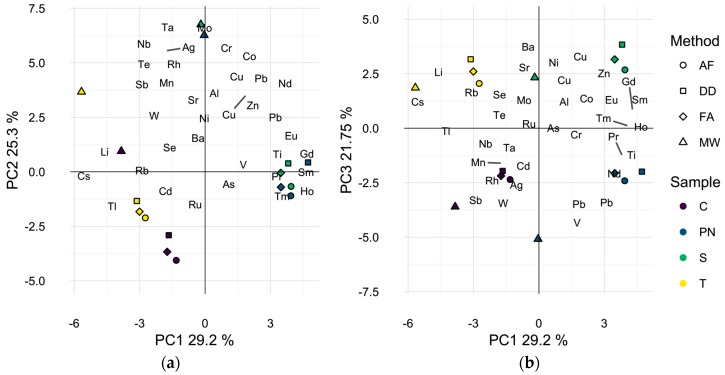
Principle component analysis biplot of wine sample scores and analytes found to differ significantly by preparation method. (**a**) First two dimensions (54.5% of total variance); (**b**) first and third dimensions (51.0% of total variance). Symbols represent different preparation methods: acidification prior to filtration (AF = ○); direct dilution (DD = □); filtration prior to acidification (FA = ◊); microwave digestion (MW = △). Wine varieties are color-coded: Chardonnay (C = purple), Pinot noir (PN = blue), Syrah (S = green), and Tempranillo (T = yellow).

The elemental profile of each wine by preparation method was also visualized by Principal Component Analysis (PCA) as a dimensionality reduction technique. PCA develops linear combinations of the variables (i.e., isotopes) that capture the maximum amount of variance in the sample data. The first three principal components (PCs) accounted for over 76% of the total variance observed in the elemental profiles of the wines (Figure 1). The first two principal components (54.5% of total variance) appear to separate microwave digested samples from other methods, Figure 1a, due to higher levels of most elements in the microwave digested samples (Table A1). Microwave digested samples for all four wines are located in the top left quadrant of Figure 1a, while all other samples group according to the wine and not the sample preparation method, indicating minimal differences in elemental composition of the direct dilution and filtration methods. Alternatively, a similar biplot of the first and third principal components (51.0% of total variance) appears to differentiate the samples by wine grape cultivar, Figure 1b, indicating that the elemental variability due to wine cultivar is smaller than due to sample preparation method, when comparing MW to the other methods.

**Figure 2:**
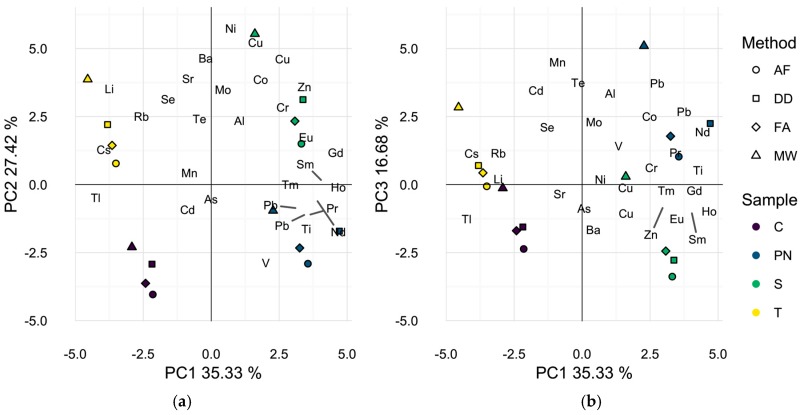
Principal component analysis biplots of wine scores and analyte loadings for each sample found to differ by preparation method with seven additional elements removed. (**a**) First two dimensions (62.8% of total variance); (**b**) first and third dimensions (52.0% of total variance). Symbols represent different preparation methods: acidification prior to filtration (AF = ○); direct dilution (DD = □); filtration prior to acidification (FA = ◊); microwave digestion (MW = △). Wine varieties are color-coded: Chardonnay (C = purple), Pinot noir (PN = blue), Syrah (S = green), and Tempranillo (T = yellow).

To better represent differences in wine elemental profiles, isotopes that showed no significant sample effect by ANOVA with the method blanks were excluded and a second PCA was performed, Figure 2a,b. Five elements, ^93^Nb, ^101^Ru, ^103^Rh, ^107^Ag, and ^181^Ta were excluded as a result of this analysis. Additionally, ^123^Sb and ^182^W were omitted, since method blank concentrations exceeded levels encountered in the samples by at least one method. Interestingly, the first two principal components (62.8% of total variance) in this analysis separated the samples primarily by wine grape cultivar with each wine prepared by all methods localized in one of the four quadrants of the data space. This result suggests that all preparation methods used may adequately differentiate these wines by elemental composition and explains the previously reported successful applications of different sample preparation methods in discriminant analyses of wine on the basis of geographic origin [2,4,11,23,27]. Nevertheless, closer inspection of the biplot of the wines in the first and third dimensions (52.0% of total variance), Figure 2b resembles Figure 1a in that the microwave digested wine samples appear to be separated in the data space from wines of the same grape cultivar, prepared by DD, AF, or FA. It is likely that the omission of ^123^Sb and ^182^W contributed to the less dramatic spatial separation by preparation strategy in Figure 2b as average wine concentrations were significantly higher in MW samples than all other methods. However, the same general trend is observed.

### 2.3. Spike Recovery

**Figure 3:**
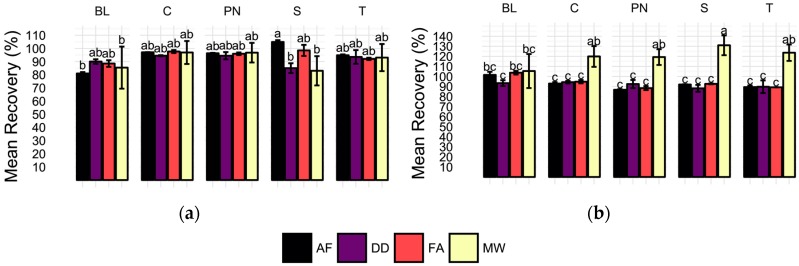
Average (**a**) ^65^Cu and (**b**) ^206^Pb spike recoveries (%) for each sample type (BL = method blank, C = Chardonnay, PN = Pinot noir, S = Syrah, T = Tempranillo) and preparation method (AF = acidification prior to filtration, DD = direct dilution, FA = filtration prior to acidification, MW = microwave-assisted digestion). Error bars represent one standard deviation. Means not sharing a letter (a, b, c) are significantly different by Tukey’s HSD’s. Recoveries calculated as the difference of concentration in the spiked sample and average unspiked sample concentration divided by the spike level, expressed as percentage.

Recovery of 100 µg/L ^65^Cu additions to each wine sample and method blank was expected to vary with respect to sample preparation method after ANOVA revealed a significant sample preparation method effect for ^65^Cu. However, recoveries were not found to be significantly different by ANOVA, Figure 3a. Comparison of mean recoveries of each method by wine cultivar shows no significant differences among methods for all samples except the Syrah (S), in which AF yielded significantly higher recoveries than both DD and MW. Recoveries in MW samples were the least precise with an average RSD of 11.98 (%) per sample. In contrast, RSDs of the direct methods were all below four percent. Despite differences in recovery precision among the methods, it is important to note that total ^65^Cu recoveries for all methods ranged from 80 to 105% and were not found to be significantly different by Tukey’s Honestly Significant Differences (HSDs).

Similar to ^65^Cu, ^206^Pb determination was found to vary with preparation method in the wine samples as determined by ANOVA. Recovery of 5 µg/L ^206^Pb spikes in wines and method blanks reveals significant differences for all wines when averaged by sample preparation method and wine cultivar, Figure 3b. Average method blank recoveries did not differ significantly by preparation method. In all four wine samples, significantly higher recoveries are observed for MW samples than the direct methods, which did not differ from one another in any samples. Additionally, MW samples were found to have the most variation in all sample types, with an average RSD of 9.1%, although this is an acceptable level in this analysis. In concordance, averaging of recoveries by preparation treatment shows significantly higher recoveries in MW samples (Tukey’s HSD used for means comparisons; data not shown). The lack of significantly different recoveries in spiked method blanks indicates that any matrix effects observed are unlikely to be the result of ethanol content alone.

### 2.4. Cu Isotope Dilution

**Figure 4:**
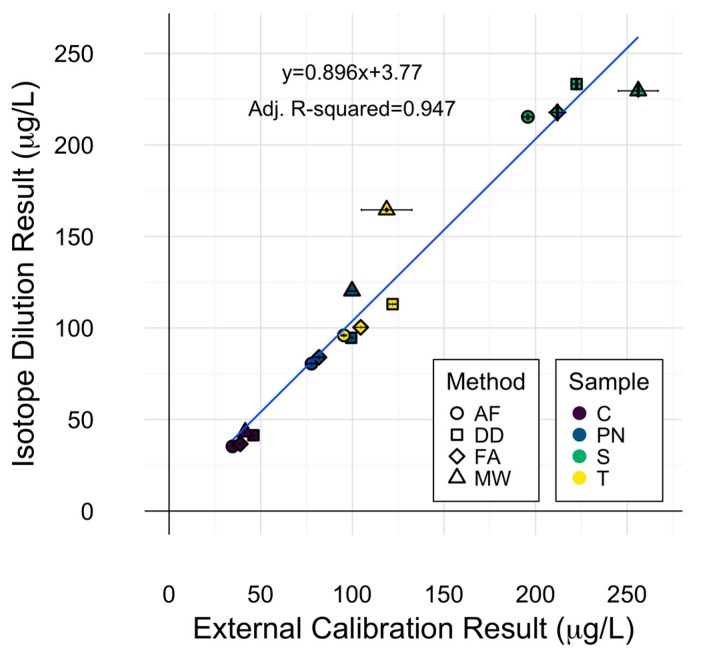
Comparison of external calibration and isotope dilution for wine copper determination. Error bars represent one standard deviation from the mean (*n* = 3). Symbols represent different preparation methods: acidification prior to filtration acidification prior to filtration (AF = ○); direct dilution (DD = □); filtration prior to acidification (FA = ◊); microwave digestion (MW = △). Wine varieties are color-coded: Chardonnay (C = purple), Pinot noir (PN = blue), Syrah (S = green), and Tempranillo (T = yellow).

The use of stable isotope standards for spiking experiments allowed for the exploration of applying isotope ratios as a means of quantitation. Internal calibration via isotope dilution was explored in this experiment and compared to external calibration by measuring the ^63^Cu:^65^Cu instrumental response ratio. ANOVA of copper concentrations of each sample with calibration method as an additional source of variation (i.e., quantitation method was either external or internal), did not reveal significant differences due to calibration technique. The high degree of agreement between calibration techniques is represented by the linear fit depicted in Figure 4, in which a slope of 1 would indicate a perfect agreement. Isotope dilution results were within 12% of external calibration results for all samples within each preparation method, except for PN and T samples prepared via microwave digestion, in which isotope dilution yielded higher concentrations than external calibration. This could be explained by the ability of isotope dilution to account for analyte loss during sample preparation [26].

Less variation in quantitation was observed with isotope dilution (0.78% RSD) than external calibration (2.44% RSD) on average, though both calibration strategies were highly precise. These results are in accordance with similar findings for Pb isotope dilution ICP-MS analysis of wine [14,24]. Inspection of the means of isotope dilution results by each sample preparation method displayed a similar pattern to that of ^63^Cu determination by external calibration, with averaged wine concentrations by each method decreasing in the order MW > DD > FA > AF. This is a reminder that the preparation method effect remained significant in the ANOVA for determination of copper concentration using isotope dilution and serves as an isolated protection against calibration errors.

The lack of significant difference between calibration strategies demonstrates the viability of isotope dilution for wine copper ICP-MS analysis. This exploration showed comparable accuracy to external calibration without full consideration of sources of systemic error in ID measurements such as mass bias. Although isotope dilution may not be feasible for multi-element analysis, internal calibration should be considered in single-element wine analysis by plasma source technologies.

## 3. Conclusions

All direct preparation methods (i.e., direct dilution (DD), acidification prior to filtration (AF), and filtration prior to acidification (FA)) showed a high degree of similarity in measured elemental profiles for the wines analyzed, while significantly higher measured concentrations were apparent for several analytes with micowave assisted digestion (MW). The precision for the wine elemental profiles was also lower for MW digestion compared to DD, AF, and FA as determined by the spike recovery experiments. These results indicate no advantage of MW digestion as a sample preparation protocol for wine ICP-MS analysis; overall the additional preparation steps and use of reagents requred for MW digestion increase the risk of compromising sample integrity. Filtration treatments, AF and FA, with 0.45 µm PTFE filters did not yield substantial differences in overall elemental profiles in comparison with DD. However, if elements ^7^Li, ^59^Co, ^63^Cu, ^85^Rb, ^88^Sr, ^133^Cs, ^182^W, ^205^Tl, ^206^Pb, and ^208^Pb are of interest, filtration prior to analysis is not recommended due to the potential for retention of organic metal species. Wines are often passed through 0.45 µm filters prior to bottling to remove remaining particulates, and as shown in this study, this practice may alter the elemental signature in comparison to the same unfiltered wine. This could provide further evidence of the impact of winemaking practices on wine elemental profiles. Based on these results, we find DD prior to analysis to offer the best compromise between ease of use and result accuracy and precision. It is important to note, that all preparation strategies tested here were able to differentiate the wines tested and individually each preparation method may be applied for in-depth investigations. However, in the interest of monitoring toxic elements and for authenticity validation, the data acquired via different sample preparation methods may not be directly comparable and careful consideration of the sample preparation method is necessary when evaluating results from different studies. Finally, copper determination in wine using a simplified isotope dilution model yielded comparable results to those of external calibration for all preparation methods, and offers a robust avenue to mitigate challenges in wine elemental analysis by ICP-MS.

## 4. Materials and Methods

### 4.1. Reagents and Samples

Nitric acid (67–70%; ultrapure) was purchased from J.T. Baker (Center Valley, PA, USA). Ultrapure water (UPW) (18 MΩcm, EMD Millipore, Bellerica, CA, USA) and 200 proof ethanol (Koptec, King of Prussia, PA, USA) were used for matrix matching of calibration standards and method blanks. Calibration standards were prepared by diluting multi-element standard mix 1 (Ce, Dy, Er, Eu, Gd, Ho, La, Lu, Nd, Pr, Sc, Sm, Tb, Th, Tm, Y, Yb), 2A (Ag, Al, As, Ba, Be, Ca, Cd, Co, Cr, Cs, Cu, Fe, Ga, K, Li, Mg, Mn, Na, Ni, Pb, Rb, Se, Sr, Tl, U, V, Zn), 3 (Au, Hf, In, Pb, Pt, Rh, Ru, Sb, Sn, Te), 4 (B, Ge, Mo, Nb, P, Re, S, Si, Ta, Ti, W, Zr), as well as ^65^Cu and ^206^Pb stable isotope standards used in spiking experiments, and were all purchased from SPEX CertiPrep (Metuchen, NJ, USA).

Chardonnay (C), Pinot Noir (PN), Syrah (S), and Tempranillo (T) wine samples, unfiltered and not fined, with 12–15% ethanol content were supplied from the on-campus winery at UC Davis (Davis, CA, USA). Method blanks for each sample preparation method were prepared by following each preparation procedure with a 12% ethanol solution. Recovery experiments involved gravimetrically spiking aliquots of each wine and method blank sample with 100 µg/L ^65^Cu and 5 µg/L ^206^Pb stable isotope standards. Spiked samples were vortexed and prepared in triplicate by each method.

### 4.2. Sample Preparation

All unfiltered wine samples (50 mL) were centrifuged at 4 °C at 5000× *g* for 10 min before preparation for analysis via all methods evaluated. All samples and blanks were evaluated by all methods in triplicate, including the use of fresh, unused filters for each replication where applicable. For sample preparation treatments were evaluated:

1. *Direct Dilution* (DD): samples were diluted 1:3 with 5% HNO_3_ (*v*/*v*) prior to analysis. Method blanks contained 4% ethanol and 5% HNO_3_ (*v*/*v*).
2. *Filtration prior to acidification* (FA): A 7 mL aliquot of sample was filtered through an Agilent Captiva Premium syringe filter (PTFE, 15 mm, 0.45 µm; Wilmington, DE, USA), then an aliquot of each filtered sample was diluted 1:3 with 5% HNO_3_ (*v*/*v*) prior to analysis. Method blank was the same as for DD.
3. *Acidification prior to filtration* (AF): A 15 mL aliquot of diluted sample (1:3 with 5% HNO_3_ (*v*/*v*)) was filtered through an Agilent Captiva Premium syringe filter (PTFE, 15 mm, 0.45 µm) prior to analysis. Method blank was the same as for DD.
4. *Microwave-assisted acid digestion* (MW): 2 × 1 mL concentrated HNO_3_ was added to 2 mL of sample in PTFE microwave tubes that were capped and digested using a Milestone UltraWAVE microwave system (Sorisole, Italy) according to manufacturer’s recommendations (20 min ramp to 240 °C and 150 bar, 15 min hold; E = 1500 W). Two milliliter aliquots of digested samples were then diluted 1:5 with ultrapure water prior to analysis. PTFE microwave vessels were cleaned using a cleaning program (15 min ramp to 230 °C and 150 bar, 5 min hold; E = 1500 W) with concentrated nitric acid according to manufacturer’s recommendations, subsequently rinsed (5% HNO_3_ and ultrapure water) and allowed to dry between digestions.

### 4.3. ICP-MS Analysis

A triple quadrupole-ICP-MS/MS 8800 from Agilent Technologies (Santa Clara, CA, USA) with a quartz double-pass spray chamber (held at 4 °C) and borosilicate micromist nebulizer (nebulizer pump speed at 0.1 rps) was used for all measurements. Helium (99.999% pure, Airgas, Sacramento, CA, USA) in the collision/reaction cell was used for all elements at a flow rate of 5 mL/min except for Li, Mo, and Te which were measured in no gas mode, and As and Se which were measured in high-energy helium mode with a 10 mL/min gas flow rate [15]. RF power and matching power were 1.5 kW and 1.8 V, respectively. The sampling depth was set at 10 mm, with an Ar (99.9999% pure, Airgas) carrier gas flow of 1.05 L/min. Most elements were monitored both without and with helium in the collision cell, and several isotopes were measured for non-monoisotopic elements, with 100 sweeps per each of three replicates. The IS mix containing 1 µg/L ^6^Li, ^45^Sc, ^72^Ge, ^89^Y, ^115^In, ^159^Tb, ^209^Bi in 1% HNO_3_ (Agilent, Santa Clara, CA, USA) was constantly fed into and mixed with the sample stream before entering the nebulizer, using a mixing tee (sample tubing inner diameter 1.02 mm, IS tubing inner diameter 0.25 mm).

Isotopes were selected based on the absence of possible interferences as well as manufacturer recommendations. Quality control consisted of the repeated analysis of the 10 µg/L calibration standard of each calibration series approximately every fifteen samples during analysis. Quantified isotopes were found to be accurate (within 20%) and precise (%RSD < 20%) across all analytical runs in replications of the 10 µg/L calibration standards used as quality control material. Limits of detection (LODs) were determined as the critical value (*p* ≤ 0.05) from the one-tailed t-distribution times the standard deviation of six measurements (*t* = 3.365) of the matrix-matched calibration blank per analytical run for the direct methods and eight measurements (*t* = 2.998) for analysis of microwave digested samples [28]. Reported LODs for the direct methods are the average obtained from two analytical runs. Calibration blanks were measured throughout each sequence batch as continuing calibration blanks (CCB).

Elements were quantified using matrix-matched calibration standards, 4% ethanol and 5% HNO_3_ for the DD, FA, and AF treatments, and 5% HNO_3_ for the MD treatments. The quantified isotopes were ^7^Li, ^27^Al, ^47^Ti, ^51^V, ^52^Cr, ^55^Mn, ^59^Co, ^60^Ni, ^63^Cu, ^65^Cu, ^66^Zn, ^71^Ga, ^75^As, ^78^Se, ^85^Rb, ^88^Sr, ^93^Nb, ^98^Mo, ^101^Ru, ^103^Rh, ^107^Ag, ^111^Cd, ^123^Sb, ^125^Te, ^133^Cs, ^137^Ba, ^140^Ce, ^141^Pr, ^146^Nd, ^147^Sm, ^153^Eu, ^157^Gd, ^163^Dy, ^165^Ho, ^166^Er, ^169^Tm, ^172^Yb, ^181^Ta, ^182^W, ^205^Tl, ^206^Pb, ^208^Pb, and ^238^U.

#### 4.3.1. External Calibration

Six point calibrations between 0 and 500 µg/L were prepared separately for all four multielement standard mixes using matrix matched solutions, 4% ethanol and 5% HNO_3_ for DD, FA, and AF treatments, and 5% HNO_3_ for MW. A separate matrix matched calibration between 0 and 500 µg/L was realized containing ^65^Cu and ^206^Pb stable isotope standards. All calibration standards were prepared gravimetrically.

#### 4.3.2. Isotope Dilution

Copper in the spiked samples was also determined using isotope dilution calculations and compared to results obtained by external calibration. The total ^63^Cu concentration in the spiked samples was calculated from the measurement of the instrument response ratio of ^63^Cu:^65^Cu using Equation (1):

(1) [Cu63]=[CuSpike65]×(mspikemsample)×(WspikeWsample)×(R×A65−A63B63−R×B65),

in which *R* is the instrument response ratio, $m_{spike}$ is the mass of the spike solution added to the sample, $m_{sample}$ is the mass of the sample, *W_spike_* is the atomic weight of Cu in the sample, *W_sample_* is the atomic weight of *Cu* in the spike, *A*_65_ and *A*_63_ are the respective abundances of ^65^*Cu* and ^63^*Cu* in the spike, and B_63_ and B_65_ are the natural abundances of ^63^*Cu* and ^65^*Cu*, 0.6915 and 0.3085, respectively [29]. Assumptions in this exploratory calculation include the use of manufacturer’s certified isotopic abundance values, *A*_65_ and *A*_63_, atomic weight in the spike, *W_spike_*, as well as the omission of instrumental mass bias correction.

### 4.4. Statistical Analysis

For all analyses, statistical significance was evaluated at (*p* ≤ 0.05). For statistical computation purposes, all values below detection limits for an element were replaced with one-half the detection limit for that element [30]. Multivariate Analysis of Variance (MANOVA) was carried out to determine whether an overall sample preparation method effect was present before univariate Analysis of Variance (ANOVA) for the main effects *sample*, *replicate*, and *method* was applied for each element. Mean comparisons by preparation method were made using Tukey’s Honestly Significant Differences (HSD) for elements found to vary significantly by ANOVA. Principal Component Analysis (PCA) of sample analyte means was carried out as an unsupervised dimensionality reduction technique using a correlation matrix to account for differences in concentration range. All statistical analyses were carried out in RStudio (version 1.0.136, Boston, MA, USA; www.rstudio.org), an open source interface for the open source statistical programming language R (version 3.0.2, Vienna, Austria; www.rproject.or), with the additional packages *FactoMineR*, *agricolae*, and *ggplot2* [31,32,33].

## Figures and Tables

**Table 1:** Instrumental detection limits (LODs) and average (*n* = 3) method blank concentrations of all isotopes. Values in italic are below the limit of quantitation (LOQ).

Isotope	Mode ^1^	LOD ^2^		Method Blank Concentration [µg/L]			
		AF-DD-FA	MW	AF	DD	FA	MW
^7^Li	NG	0.103	0.029	1.23	1.77	1.50	0.962
^27^Al	He	0.802	0.988	<LOD	1.84	<LOD	13.7
^47^Ti	He	0.236	0.174	0.611	<LOD	<LOD	2.27
^51^V	He	0.010	0.008	0.013	0.020	0.015	0.029
^52^Cr	He	0.220	0.049	<LOD	1.04	<LOD	5.07
^55^Mn	He	0.068	0.041	<LOD	0.772	<LOD	20.1
^59^Co	He	0.005	0.003	<LOD	0.026	<LOD	1.36
^60^Ni	He	0.086	0.042	<LOD	0.600	<LOD	5.22
^63^Cu	He	0.049	0.042	<LOD	<LOD	<LOD	0.248
^65^Cu	He	0.018	0.010	<LOD	<LOD	<LOD	0.045
^66^Zn	He	0.280	0.184	<LOD	<LOD	<LOD	1.16
^71^Ga	He	0.005	0.003	0.007	0.006	0.010	<LOD
^75^As	HEHe	0.013	0.011	0.056	0.154	0.116	0.106
^78^Se	HEHe	0.080	0.018	<LOD	<LOD	<LOD	0.280
^85^Rb	He	0.043	0.065	0.467	0.558	0.279	0.130
^88^Sr	He	0.021	0.020	0.065	<LOD	<LOD	0.050
^93^Nb	He	0.007	0.007	<LOD	0.018	<LOD	0.524
^98^Mo	NG	0.075	0.017	<LOD	<LOD	0.099	1.96
^101^Ru	He	0.008	0.009	0.009	<LOD	<LOD	<LOD
^103^Rh	He	0.002	0.001	<LOD	<LOD	<LOD	0.034
^107^Ag	He	0.015	0.002	<LOD	<LOD	<LOD	0.138
^111^Cd	He	0.007	0.009	0.040	<LOD	0.040	<LOD
^123^Sb	He	0.043	0.012	<LOD	0.060	<LOD	0.732
^125^Te	NG	0.003	0.003	<LOD	0.007	0.004	0.005
^133^Cs	He	0.018	0.014	0.029	0.033	0.074	0.033
^137^Ba	He	0.041	0.041	<LOD	<LOD	<LOD	0.124
^140^Ce	He	0.002	0.004	0.003	<LOD	<LOD	<LOD
^141^Pr	He	0.0005	0.003	<LOD	0.003	<LOD	<LOD
^146^Nd	He	0.002	0.003	0.005	0.002	0.002	0.003
^147^Sm	He	0.002	0.002	0.002	0.004	<LOD	<LOD
^153^Eu	He	0.0005	0.003	<LOD	0.001	<LOD	<LOD
^157^Gd	He	0.001	0.004	<LOD	0.004	<LOD	<LOD
^163^Dy	He	0.001	0.005	<LOD	<LOD	<LOD	<LOD
^165^Ho	He	0.0004	0.003	0.001	0.0004	<LOD	<LOD
^166^Er	He	0.001	0.003	<LOD	0.003	<LOD	<LOD
^169^Tm	He	0.001	0.002	<LOD	0.001	<LOD	<LOD
^172^Yb	He	0.001	0.003	0.008	0.004	0.005	0.005
^181^Ta	He	0.022	0.033	<LOD	0.032	<LOD	1.94
^182^W	He	0.117	0.013	<LOD	0.189	0.122	1.27
^205^Tl	He	0.002	0.003	0.005	0.007	0.008	<LOD
^206^Pb	He	0.009	0.003	<LOD	0.140	<LOD	0.030
^208^Pb	He	0.007	0.004	0.017	0.130	<LOD	0.043
^238^U	He	0.004	0.001	0.009	<LOD	0.009	<LOD

^1^ Use of CRC in no gas (NG), helium (He), or high energy helium (HEHe) mode; ^2^ LOD = *t* × SD; *n* = 6, *t* = 3.365 (AF—Acidification prior to Filtration, FA—Filtration prior to Acidification, DD—Direct Dilution) and *n* = 8, *t* = 2.998 (MW—Microwave Digestion) calibration blanks. LOQ = 10 × SD of calibration blanks.

## Appendix A

**Table A1:** Mean wine concentrations (µg/L) ± standard error of all analytes. Letters denote an analyte with a significant method effect by ANOVA (*p* ≤ 0.05), means not sharing a letter for an isotope are significantly different by Tukey HSD’s (AF: Acidification prior to Filtration; DD: Direct Dilution; FA: Filtration prior to Acidification; MW: Microwave Digestion).

Isotope	AF	DD	FA	MW
^7^Li	12.3 ± 0.209 b	18.8 ± 0.483 a	14.3 ± 0.614 b	20 ± 1.25 a
^27^Al	247 ± 6.97 c	281 ± 9.13 b	267 ± 12.3 b	325 ± 13.2 a
^47^Ti	6.04 ± 0.577 ab	5.02 ± 0.736 ab	6.58 ± 0.621 a	4.7 ± 2.04 b
^51^V	1.16 ± 0.033 b	1.33 ± 0.061 a	1.26 ± 0.05 ab	1.36 ± 0.12 a
^52^Cr	14.0 ± 0.33 b	16.1 ± 0.308 ab	15.1 ± 0.564 ab	20.8 ± 6.15 a
^55^Mn	1100 ± 24.4 d	1190 ± 27.7 b	1150 ± 26.3 c	1310 ± 41.2 a
^59^Co	4.26 ± 0.074 c	5.23 ± 0.103 b	4.5 ± 0.103 c	5.63 ± 0.139 a
^60^Ni	30.0 ± 0.44 c	36.4 ± 0.356 a	32.8 ± 1.06 b	36.9 ± 1.99 a
^63^Cu	101 ± 1.5 b	122 ± 1.36 a	109 ± 2.74 b	129 ± 6.65 a
^65^Cu	31.9 ± 0.47 b	35.5 ± 0.46 b	35.1 ± 0.979 b	44.0 ± 2.28 a
^66^Zn	634 ± 8.68 c	682 ± 8.25 b	686 ± 17 b	717 ± 25.1 a
^71^Ga	0.109 ± 0.023	0.107 ± 0.025	0.113 ± 0.03	0.125 ± 0.125
^75^As	0.954 ± 0.032 c	1.65 ± 0.525 a	1.25 ± 0.095 b	1.03 ± 0.108 bc
^78^Se	1.42 ± 0.066 b	2.05 ± 0.457 a	1.99 ± 0.557 a	1.85 ± 0.533 ab
^85^Rb	1740 ± 28.3 b	1970 ± 49.8 a	1690 ± 11.9 b	1950 ± 46.7 a
^88^Sr	578 ± 7.13 b	784 ± 13.4 a	576 ± 6.95 b	779 ± 19.9 a
^93^Nb	<LOD b	<LOD b	<LOD b	0.346 ± 0.098 a
^98^Mo	1.52 ± 0.052 b	1.28 ± 0.044 b	1.64 ± 0.057 b	3.18 ± 0.524 a
^101^Ru	<LOD b	<LOD b	0.012 ± 0.005 a ^1^	<LOD b
^103^Rh	<LOD b	0.004 ± 0.003 b ^1^	<LOD b	0.019 ± 0.009 a
^107^Ag	<LOD b	<LOD b	0.016 ± 0.002 b ^1^	0.091 ± 0.054 a
^111^Cd	0.189 ± 0.01 ab	0.183 ± 0.006 ab	0.198 ± 0.01 a	0.177 ± 0.017 b
^123^Sb	0.188 ± 0.015 b	0.11 ± 0.013 b ^1^	0.202 ± 0.012 b	0.6 ± 0.296 a
^125^Te	0.018 ± 0.003 c	0.037 ± 0.009 b	0.033 ± 0.016 bc	0.061 ± 0.031 a
^133^Cs	3.36 ± 0.075 c	4.01 ± 0.094 b	3.37 ± 0.112 c	4.42 ± 0.14 a
^137^Ba	167 ± 1.94 b	155 ± 2.19 c	170 ± 2.86 b	177 ± 5.15 a
^140^Ce	0.047 ± 0.002	0.047 ± 0.003	0.049 ± 0.002	0.047 ± 0.008
^141^Pr	0.004 ± 0.001 b	0.008 ± 0.001 a	0.004 ± 0.001 b	<LOD b
^146^Nd	0.034 ± 0.006 b	0.029 ± 0.005 b	0.034 ± 0.005 b	0.048 ± 0.018 a
^147^Sm	0.011 ± 0.003 a	0.011 ± 0.006 a	0.01 ± 0.004 a	0.003 ± 0.003 b ^1^
^153^Eu	0.003 ± 0.002 ab	0.004 ± 0.001 a	0.003 ± 0.001 b	<LOD b
^157^Gd	0.013 ± 0.003 a	0.016 ± 0.004 a	0.012 ± 0.003 a	0.006 ± 0.004 b ^1^
^163^Dy	0.024 ± 0.005	0.019 ± 0.004	0.023 ± 0.003	0.018 ± 0.008
^165^Ho	0.006 ± 0.001 a	0.006 ± 0.001 a	0.006 ± 0.001 a	<LOD b
^166^Er	0.027 ± 0.003	0.024 ± 0.003	0.024 ± 0.002	0.022 ± 0.006
^169^Tm	0.004 ± 0.001 b	0.007 ± 0.001 a	0.003 ± 0.001 b	<LOD c
^172^Yb	0.049 ± 0.008	0.045 ± 0.005	0.047 ± 0.007	0.044 ± 0.008
^181^Ta	<LOD b	0.18 ± 0.166 b	0.046 ± 0.035 b ^1^	1.06 ± 0.429 a
^182^W	0.257 ± 0.041 c ^1^	0.509 ± 0.114 b	0.298 ± 0.033 c ^1^	0.831 ± 0.436 a
^205^Tl	0.143 ± 0.004 b	0.189 ± 0.005 a	0.15 ± 0.006 b	0.182 ± 0.016 a
^206^Pb	0.612 ± 0.019 c	0.738 ± 0.021 b	0.538 ± 0.013 c	0.963 ± 0.051 a
^208^Pb	2.36 ± 0.06 b	2.89 ± 0.06 a	2.26 ± 0.032 b	3.13 ± 0.155 a
^238^U	0.025 ± 0.002	0.029 ± 0.003	0.026 ± 0.001	0.031 ± 0.004

^1^ Value below quantitation limit (LOQ): LOQ = 10 × SD.
